# A Singular Case of Delirium Relieved by Chloroform

**Published:** 1853-09

**Authors:** C. J. Pope

**Affiliations:** Alabama


*A Singular Case of Delirium Relieved by Chloroform.* By C. J. Pope, M. D., of Alabama.

What I propose on the present occasion is, to direct attention to the internal administration of chloroform in all cases of exaltation of vital action, dependent on nervous excitability.

The effects of this remedy in the case recorded below, although a solitary one, were so marked and salutary, that my mind was brought to the conclusion that in all similar cases its effects would be no less striking.

About the first of February I was called in haste to see a lad, of about fourteen years of age, who, immediately on rising from his bed, at a very early hour in the morning, and before it was entirely light, went under the dwelling of his father for the purpose of getting the eggs out of a hen's nest, in a hole of considerable depth, scratched out by the dogs. On getting into the hole, which was in depth about two-thirds his entire length, the hen which he had not supposed to be on the nest at that early hour, flew into his face, and this circumstance, together with that of his hold breaking, on attempting quickly to recover himself, so frighted him that spasms of the most violent character were the result. I saw him in half an hour after this happened, and at once opened the temporal artery, and bled him two ounces, which however had no effect in controlling the spasms. In a few minutes after the blood was stopped, I gave him an injection of tinct. of lobelia. In five minutes the violence of the spasms seemed to be slightly controlled, but returned again in ten minutes more. The lobelia was again administered with the same partial effect, which lasted about the same length of time. Foiled in all my efforts thus far to arrest the spasms even temporarily, I had recourse to many other antispasmodics, but to no effect. The spasms continued for thirty-six hours, at the end of which time they passed off, and left him in a most singular state of delirium, which lasted seven or eight days without a lucid interval.

Large doses of opium and camphor would partially quiet him for an hour or two, but the delirium invariably returned. Finally, having given up all hope of his recovery, I resolved upon the following prescription: Aquæ camphoræ ℥ii, tinct. valerian ℥ii, chloroform ℥i, mixed. Of this I gave him a tablespoonfull, and in five minutes I perceived indications of quietude. I waited one hour and a half, at the expiration of which time I found symptoms of returning delirium. I then gave him a second and rather larger dose. In ten minutes he was quiet, in twenty-five minutes I had the satisfaction of seeing him in a fine sleep, which lasted all night, (it being then about ten o'clock,) and out of which he awoke on the following morning, entirely restored in mind, without any consciousness of what had transpired during the eight days of his illness.

His convalesence was prompt, and his recovery perfect.

